# Extracellular Vesicles in Human Oogenesis and Implantation

**DOI:** 10.3390/ijms20092162

**Published:** 2019-05-01

**Authors:** Francesca Andronico, Rosalia Battaglia, Marco Ragusa, Davide Barbagallo, Michele Purrello, Cinzia Di Pietro

**Affiliations:** 1 Department of Biomedical and Biotechnological Sciences, Biology and Genetics Section G. Sichel, University of Catania, 95123 Catania, Italy; francesca.andronico@gmail.com (F.A.); mragusa@unict.it (M.R.); dbarbaga@unict.it (D.B.); purrello@unict.it (M.P.); 2 Oasi Research Institute—IRCCS, 94018 Troina, Italy

**Keywords:** MicroRNAs, Extracellular vesicles, Exosomes, Microvesicles, Oocyte, Blastocyst, Endometrium

## Abstract

Reproduction, the ability to generate offspring, represents one of the most important biological processes, being essential for the conservation of the species. In mammals, it involves different cell types, tissues and organs, which, by several signaling molecules, coordinate the different events such as gametogenesis, fertilization and embryo development. In the last few years, the role of Extracellular Vesicles, as mediators of cell communication, has been investigated in every phase of these complex processes. Microvesicles and exosomes, identified in the fluid of ovarian follicles during egg maturation, are involved in communication between the developing oocyte and the somatic follicular cells. More recently, it has been demonstrated that, during implantation, Extracellular Vesicles could participate in the complex dialog between the embryo and maternal tissues. In this review, we will focus our attention on extracellular vesicles and their cargo in human female reproduction, mainly underlining the involvement of microRNAs in intercellular communication during the several phases of the reproductive process.

## 1. Introduction

Cell-to-cell communication has allowed the development of multicellular organisms in which cells do not live alone but have to respond to different signals, contributing to the safety of the whole organism. In addition to direct interaction (gap junctions), autocrine, paracrine, or endocrine signaling, over the last few years, a new intercellular communication tool, mediated by Extracellular Vesicles (EVs) has emerged. Although some authors supposed that secreting EVs could represent a mechanism used by cells to leave out unnecessary molecules, their involvement in intercellular signaling, has been widely discussed [1]. By means of EVs, different molecules such as lipids, proteins and nucleic acids (DNA, mRNAs, non-coding RNAs) can be transferred from donor cells to recipient cells. Because of their involvement in physiological cellular processes and in human diseases, EVs and their cargo have attracted several studies, ranging from different fields of biology to medicine [2,3]. The detailed characterization of the different molecular cargos opened new promising applications in diagnosis and therapy: to find non-invasive markers of different pathologies, to identify new therapeutic targets and to develop innovative drug delivery systems [4,5,6].

According to their size and the biogenesis mechanisms, EVs are classified in exosomes, microvesicles and apoptotic bodies. Exosomes are nano-sized vesicles (from 40 to 100 nm in diameter), bounded by a bilayer membrane, secreted by most cell types both in physiological and pathological conditions. Exosomes derive from the endocytic compartments, in particular from Multivesicular Bodies (MVBs) and are released when the MVBs fuse with the plasma membrane by the exocytosis. Microvesicles, having a diameter of 100–1000 nm, are released by budding or shedding of the plasma membrane and then the apoptotic bodies, with the broadest range of diameters (50–5000 nm), produced by cells undergoing apoptosis [7,8,9].

Despite the growing interest in the study of EVs and their cargo, to date, the methods of separation and characterization have certainly not been ideal. Multi-step differential centrifugation and the search for specific exosome markers, such as Endosomal Sorting Complexes Required for Transport (ESCRT), and tetraspanins (CD63, CD81 and CD9), despite the limitations, remain the most widely used methods. Now, alternative techniques such as flow cytometry, Electron Microscopy (EM), western blotting, enzyme-linked immunosorbent assay, Nanoparticle Tracking Analysis (NTA), dynamic light scattering, cryo-EM, atomic force microscopy, Raman spectroscopy and resistive pulse sensing have been developed [8].

In the last few years, it has been demonstrated that EVs play a different and important role in the main phases of the reproductive process such as gametogenesis, fertilization, implantation and embryo development [10]. The birth of a new life is the result of proper communication among the cells of different tissues and organs and, in addition, depends on two different genomes. A suitable comprehension of the different molecules and mechanisms specifically involved in the different phases is not only important in basic research, but could clarify cases of unexplained infertility, improving reproductive success.

In males, the role of EVs in sperm maturation, capacitation, acrosomal reaction and fertilization has been investigated and an exhaustive review has been recently published [10]. In this paper, we will discuss the involvement of EVs in human female reproduction, focusing our attention, particularly, on EVs in follicle maturation, implantation and early embryo development.

## 2. Follicle Growth and Oocyte Maturation

**Figure 1:**
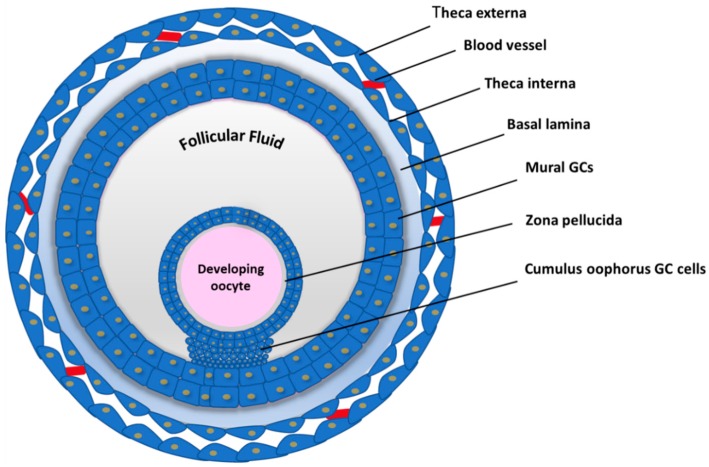
Schematic representation of the Ovarian Follicle structure.

In mammals, oogenesis is a long-lasting process that begins during embryonic life and ends with menopause. During embryonic life, the oogonia, precursors of female gametes, expand their number by mitosis, enter in meiosis as primary oocytes and become arrested in prophase I. Each oocyte is enclosed by somatic cells (pre-granulosa cells) to form a primordial follicle. At birth, the pool of primordial follicles represents the woman’s ovarian reserve. After puberty, cyclically, some primordial follicles from the prenatal pool, develop through different stages. The follicles grow, the oocyte resumes meiosis and after a careful selection, only the dominant follicle is chosen to produce the mature egg, ready for fertilization. Ovarian Follicles represent the reproductive units consisting of the oocyte, somatic cells (cumulus granulosa, mural granulosa and theca cells) and follicular fluid (FF) (Figure 1).

Follicle growth and oocyte maturation are connected by a constant exchange of signals between the somatic components and the germ cell [11]. The cross-talk between the oocyte and somatic follicular cells occurs by gap-junctions established between the oocyte and CCs, and by FF accumulated inside the follicle during maturation. FF formation is due to the infiltration of several capillaries, which provide several plasma components, fundamental for oocyte growth and to the secretory activity of the somatic follicular cells and oocyte [12]. Its composition, primarily represented by proteins, steroid hormones, enzymes, growth factors, fatty acids, cytokines and anticoagulants factors, permits intercellular communication and provides the oocyte with nutrition and allows it to mature inside the follicle [13,14,15].

In the last few years, EVs have been identified in FF and, to date, more than 30 papers have been published. The first papers, characterizing FF vesicles, focused on the biological function of exosomes, microvesicles and their molecule cargo inside the ovarian follicle. This represented an innovation if compared to other research fields, where EV physiological functions are surely less investigated than their role in human diseases. It has been demonstrated that FF EVs are involved in the regulation of pathways controlling follicular growth and hormone response as well as oocyte cytoplasmic maturation and meiosis resumption. As concerns the different molecule cargos of FF EVs, to date, microRNAs (miRNAs) and proteins have been identified; however, there are no data about mRNAs, long non-coding RNAs and circular RNAs in humans. Most of the studies have been performed on animal models that represent a very important resource in reproductive research; because of ethical considerations, it is not possible to perform functional studies on humans. Profiling studies, carried out primarily on circulating miRNAs, have revealed clear expression differences related to different female reproductive disorders confirming the role of EVs and their cargo in the development of human female gametes.

One of the first studies detecting the presence of microvesicles and exosomes in horse FF demonstrated that miRNAs packaged in EVs were present in granulosa and cumulus cells and that somatic follicular cells are able to bind and capture the vesicles, suggesting a new cell-to-cell communication inside the ovarian follicles [16]. Recently, the uptake of EVs by bovine granulosa cells and their presence in cytoplasmatic projections among the cumulus cells and the early stage oocyte have been observed, confirming the role of a signaling mediator of FF EVs. Moreover, in this paper the authors identified 280 EV miRNAs, some of them able to regulate the Insulin-like Growth Factor 2 (IGF2) pathway involved in follicle growth and in the control of steroidogenesis, by induction of aromatase expression [17].

In 2013, for the first time, by electron microscopy, microvesicles were identified in human FF and several miRNAs were detected. By using cultured KGN cell lines, the authors found miRNAs affecting steroidogenesis, regulating estradiol and progesterone concentrations [18].

A few months later, Santonocito et al. isolated and characterized exosomes from human FF by ultracentrifugation, Nanoparticle Tracking Analysis and Flow Cytometry. They found several miRNAs that were exosome cargo and in order to detect miRNAs specifically synthesized from follicular cells and not released by blood cells, they compared miRNA expression in FF with plasma from the same women and selected the miRNAs upregulated in FF. By this approach, they selected 32 exosomal miRNAs and by bioinformatic analysis found that specific FF miRNAs are able to regulate follicular development, meiotic resumption, and subsequent ovulation [19].

Experimental evidence of the FF EVs involvement in follicular maturation came from animal models. Hung et al. demonstrated that bovine follicular EVs are taken up by bovine or mouse cumulus cells and are able to induce cumulus-oocyte complex (COC) expansion by modulating the expression of genes with known functions in this process, such as Prostaglandin-endoperoxide synthase 2 (Ptgs2) and Pentraxin 3 (Ptx3) [20]. EVs coming from three stages of oocyte maturation in mares (early, mid-estrus and pre-ovulation) present dissimilar concentrations and different miRNA contents depending on the stage. Specifically, miR-125 and miR-199, probably secreted by granulosa cells and packaged in EVs, were particularly expressed in the pre-ovulatory stages, being involved in oocyte maturation and cumulus proliferation and miR-21, miR-132, and miR-212, whose expression seems to be hCG/LH-related, were identified in EVs released by granulosa cells. They probably induce granulosa cell proliferation and the maturation of the cumulus-oocyte complex [21].

Further confirmation of the important role of EVs and their miRNA cargo inside the ovarian follicle is inferred by studies comparing miRNA profiling in different conditions that impair woman’s fertility. Polycystic Ovary Syndrome (PCOS) and Premature Ovarian Failure (POF) and also lifestyle and aging are determinant conditions in ovarian dysfunction. Female reproductive aging represents the most common cause of reproductive failure. In fact, it is widely accepted that reproductive aging has a strong impact on pregnancy rate because of the reduction of the ovarian reserve and of oocyte/embryo quality [22,23]. Mares are an established model of reduced oocyte quality due to reproductive aging [24]; thus Da Silveira et al. collected EVs from young and old mares and observed miRNA expression in early follicle development, mid-estrus and pre-ovulatory follicles. They noticed that aged mares had an increased number of deregulated (DE) EV miRNAs during the three different phases. In young FF samples, there were differences in miRNA production, mostly from the early development to mid-estrus transition, whereas in old samples they observed differences in all the three stages. Some DE miRNAs in mares had been previously identified in human FF and found to be involved in the regulation of ovarian function. MiR-23a, overexpressed in older mares during the mid-estrus, is up-regulated in women with POF; miR-132, up-regulated in old mares, seems to be involved in steroidogenesis control in humans; miR-222, decreased in old mares, is related to steroidogenesis in a human granulosa-like tumor cell line in vitro [16,21,25]. In addition, up-regulation of miR-181a, miR-375 and miR-513a-3p has been found in FF of older mares. These miRNAs target the Transforming Growth Factor Beta (TGFβ), and by silencing the TGFβ pathway they can cause an altered oocyte maturation [25,26]. In 2014, Diez-Fraile et al. identified four DE miRNAs in EVs from FF comparing younger with older women. Specifically, they found miR-99b, miR-134 and miR-190b upregulated and miR-21-5p downregulated in older women. These miRNAs regulate genes involved in heparan-sulfate proteoglycan expression, carbohydrate digestion and absorption, and apoptosis and their altered regulation could affect follicle development and oocyte maturation [27]. In a recent paper, the authors demonstrated that EV miRNAs expression in FF correlates to fertilization status and embryo quality. They found that miR-92a and miR-130b over-expression is related to a negative outcome in In Vitro Fertilization (IVF) cycles and that miR-214, miR-454 and miR-888 differential expression is associated with good quality embryo [28].

**Figure 2:**
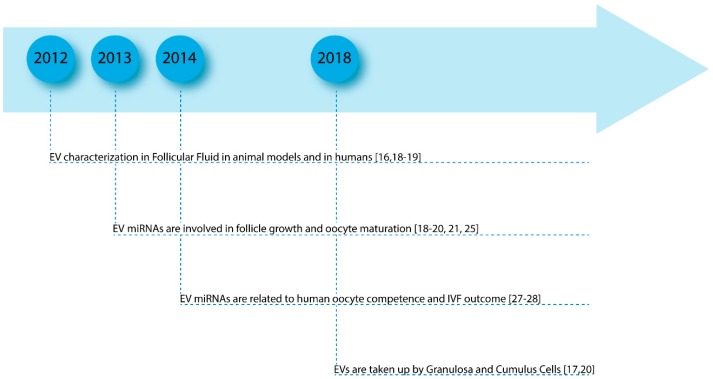
Milestones of the main significant papers about the involvement of EVs in follicle growth and their potential role in fertility disorders.

In Figure 2, we have reported the milestones of the findings and some of the most significant papers about the involvement of EVs in follicle growth and oocyte maturation and their potential role in fertility disorders (Figure 2).

## 3. Implantation

**Figure 3:**
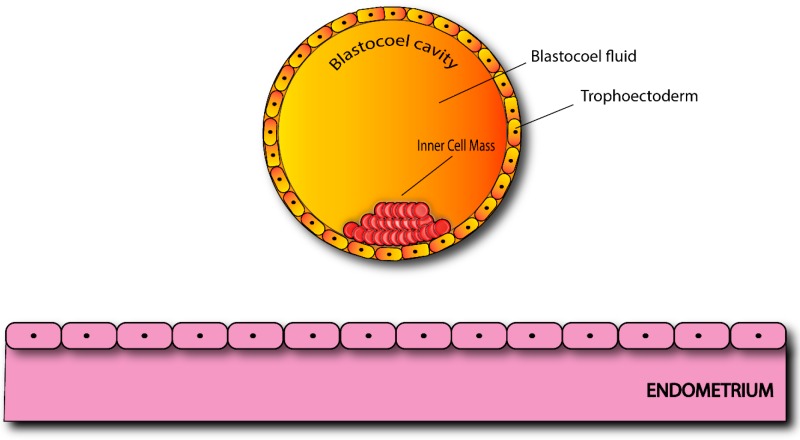
Section of a human blastocyst ready for implantation.

In humans, on the 5th day after fertilization, the embryo, hosted by the uterus, has reached the blastocyst stage and is ready for implantation. At this stage, embryo cells are differentiated into the Inner Cell Mass (ICM) and Trophectoderm Lineages (TE), and have secreted a fluid to create a fluid-filled cavity, inside the blastocyst, the blastocoel (Figure 3).

ICM represents the Embryonic Stem Cells (ESCs) giving rise to all of the cell types of the new organism. TE, interacting with maternal tissues, develops in extraembryonic membranes and placenta. On the other hand, the endometrium is at a specific window, called Window of Implantation (WOI), and is able to receive the blastocyst providing a suitable environment for embryo implantation and development [29]. Successful implantation depends on the blastocyst binding to the endometrium and the dialog between the two different compartments. Of course, many molecules are involved in this dialog: integrins, matrix-degrading enzymes, cytokines and chemokines, produced in response to ovarian steroid hormones to allow cell adhesion, modulate the local immune response and regulate gene expression in the embryo [29].

**Figure 4:**
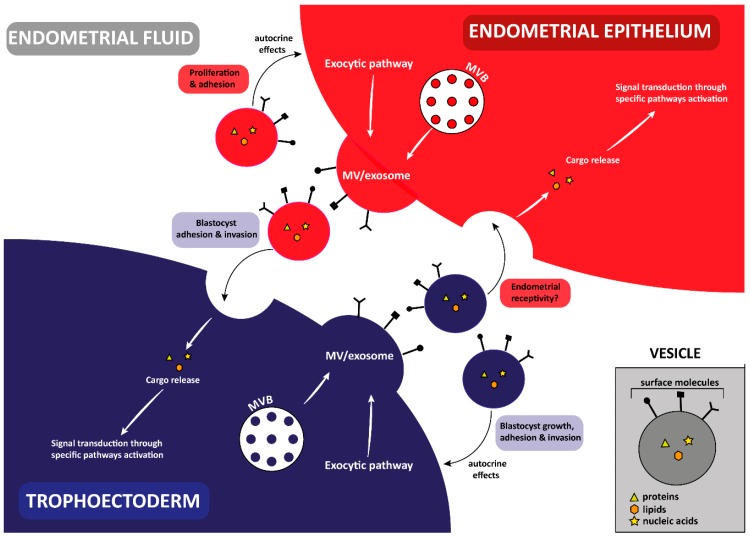
An overview of the involvement of exosomes and microvesicles (MV) in the cross-talk established between the endometrial epithelium and the trophoectoderm, probably mediated by the endometrial fluid. Released vesicles are represented with the same color of the donor cell, and the effect on the recipient cell is indicated in the colored-boxes.

Exosomes and microvesicles have recently been isolated from both the embryonic and maternal side and different papers described the export of proteins, lipids and miRNAs as EV cargo able to modulate the complex dialog from the embryo to the mother, and vice versa. In vivo, EVs have been found in uterine fluid and, in vitro, in the culture medium, released from endometrial cancer cell lines (ECC1), which represent a valuable model to study endometrial epithelial cells being estrogen and progesterone responsive. Human embryos, obtained from IVF cycles, are able to secrete exosomes and microvesicles in culture medium and it has been demonstrated that embryo EVs are taken up by primary endometrial cells. In light of this evidence, even if different aspects should be better studied, it seems to be clear that EVs perform a basic role in human implantation mediating the dialog between endometrium and embryo [30,31]. Moreover, accurate characterization of their cargo could provide useful information about the quality of the embryo and endometrial receptivity (Figure 4).

In 2012, Braundmeier et al. demonstrated that Human uterine epithelial cells (HESs) secrete EVs containing the glycosylated transmembrane protein Extracellular Matrix Metalloproteinase Inducer (EMMPRIN), which stimulates metalloproteinase production by human uterine fibroblast cells and coordinates extracellular matrix remodeling [32]. Comparing miRNA expression in ECC1 cells and the derived vesicles, Ng et al. found 214 miRNAs common to EVs and cells, 13 specifically sorted in EVs and 5 miRNAs present only in cells. Bioinformatics analysis revealed that EV miRNAs were involved in the regulation of several KEGG pathways, some of which are implicated in the implantation process [33]. A study by Vilella et al. identified miR-30d as an exosome-associated molecule whose expression in endometrial fluid changed across different phases of endometrial receptivity. Free and exosome-associated miR-30d was taken up by trophoblastic cells of murine embryos from the endometrial fluid, modifying the embryo transcriptome and its adhesive phenotype, as confirmed by in vitro results [34]. Similarly, several small RNAs were released in EVs from cyclic and pregnant ovine uterine luminal fluid. Among them, the authors identified 81 conserved mature miRNAs, 27 of which were differentially expressed between these two sample groups. The presence of conserved miRNAs could suggest a specific mechanism constitutively used by the uterine microenvironment to facilitate the fertilization process. In addition, the authors analyzed the protein EV cargo, and they found 8 proteins common to cyclic and pregnant ovine uterine luminal fluid EVs. Normally exosomes contain proteins whose function is associated with vesicle biogenesis and function, therefore these findings are innovative [35]. In 2016, treating hECC1 cells with estrogen or estrogen plus progesterone to simulate the normal proliferative and secretory stages of the menstrual cycle, Greening et al. isolated the released exosomes and profiled their protein cargo. As expected, they found several proteins involved in biogenesis, packaging and release of the vesicles, but interestingly, they observed a new protein set not reported in exosome databases, which is only present in exosomes from human endometrial epithelium. Among them, there were several proteins involved in cell migration and adhesion, ECM remodeling, angiogenesis, and proteins able to active Focal Adhesion Kinase (FAK) signaling. Therefore, endometrial EVs contribute to creating a suitable microenvironment for endometrial embryo interactions and, consequently, for implantation [36].

The first paper demonstrating that mammalian embryos secrete EVs in culture medium was published in 2014. By using group culture systems, the authors isolated the vesicles, identified the exosomes and found the mRNAs of pluripotency genes (Oct4, Sox2, Klf4, c-Myc, and Nanog) inside them. They suggested that the exosomes, carrying stemness signals, could act as mediators to improve the development of the co-cultured embryos [37]. As reported in a comprehensive review, in an in vitro culture environment of groups of pre-implantation embryos, autocrine and paracrine signals, represented by proteins, growth factors, metabolites and EVs, have been found. It seems that these signals stimulate embryo development [38]. In the late 20th century, in order to mimic the uterine microenvironment, and to improve embryo quality, co-culture techniques employing embryos and endometrial cells were introduced. By culturing human embryos coming from women who had undergone ICSI with cumulus cells, Bhadarka et al. obtained better quality blastocyst with a higher implantation rate [39].

In a mouse model, Desrochers et al. showed that embryonic stem cell-derived microvesicles could enhance trophoblast migration by interacting with FAK and c-Jun N-terminal kinase (JNK), and, through their cargo, could promote blastocyst implantation [40]. Interestingly, Pavani et al. recently demonstrated a reduction in apoptosis and an increase in blastocyst rate, when individual embryos are cultivated in bovine serum albumin medium supplemented with EVs coming from conditioned embryo culture medium [41]. EVs secreted by cultured embryos not only seem to improve embryo competence, exchanging staminal messages among the different embryonic cells, but could also send signals to the maternal side, supporting endometrial ability towards implantation. In a very interesting paper, Giacomini et al. showed that human pre-implantation embryos are able to secrete EVs, mainly exosomes, when cultured for IVF procedures and these embryo-derived EVs are taken up by Primary Endometrial cells in culture. EV source was confirmed according to the presence of stemness transcripts and non-classical glutaminase 2 (HLGA)-protein and revealed at different developmental stages [42].

In assisted reproductive medicine, the analysis of the embryo culture medium and its relative autocrine signal component could be used to assess embryo quality. The discovery of new biomarkers, for choosing high-quality embryos to implant, is one of the most intriguing challenges in this field. In fact, about one third of embryos, produced in vitro still fail to implant into the uterine cavity. The most frequent cause of failure is represented by aneuploidies, especially in couples of advanced reproductive age. To date, Preimplantation Genetic Screening (PGS) is often associated with the morphological evaluation of embryo quality, in order to detect euploid embryos to implant. Even though the premise behind PGS is widely accepted, the safety of the biopsy stage, involving the invasive removal of cells from the trophectoderm, are still considered critical parameters [43]. Culture medium analysis could represent an innovative, non-invasive method to assess embryo competence. Pallinger et al. proposed to use flow cytometry to test the nucleic acid component of the EVs released by the embryo in the culture media. Using Propidium Iodide (PI) staining, which specifically intercalates nucleic acids, the authors demonstrated that only the embryo releasing a low number of the stained vesicle (EVs + PI) were competent, probably because a higher presence of nucleic acid could be related to cell injury and, consequently, to embryo damage [44]. Now, among the most promising biomarkers that could be used to assess embryo competence, there are miRNAs secreted in the embryo culture medium. Specifically, miRNA profiles have been correlated with fertilization methods (FIVET vs ICSI), embryo aneuploidy and pregnancy outcome [45,46]. In another study, Abu-Halima et al. analyzed extracellular vesicle secretion and miRNA expression in the spent culture media after embryo transfer. They observed a reduced miRNA repertoire, coherent with decreased EV secretion from an embryo successfully implanted in comparison with an embryo with a negative outcome [47]. Nevertheless, the high risk of non-embryonic contaminants and the low number of recoverable vesicles represent, to date, limiting factors [48]. Another emerging possibility to evaluate embryo quality is the analysis of BF. It has recently been demonstrated that human embryos are able to secrete exosomes not only outside (in culture medium in vitro and in the endometrium in vivo), but also inside the blastocyst, in BF [49]. Moreover, the authors identified different BF miRNAs involved in cellular pathways regulating pluripotency, cell cycle, apoptosis and pathways related to preimplantation embryo development such as ECM-receptor interaction, gap junction, and the Hippo signaling pathway. The presence of exosomes in BF could explain the origin of DNA fragments found inside BF and used to detect Mendelian monogenic diseases. In order to prove the feasibility of BF EVs as potential biomarkers of embryo health, it will be necessary to develop new methodologies able to isolate the EVs from a reduced amount of fluid. This will allow their characterization and the investigation of their molecule cargo, searching for definite gene mutations or specific miRNA profiles.

**Figure 5:**
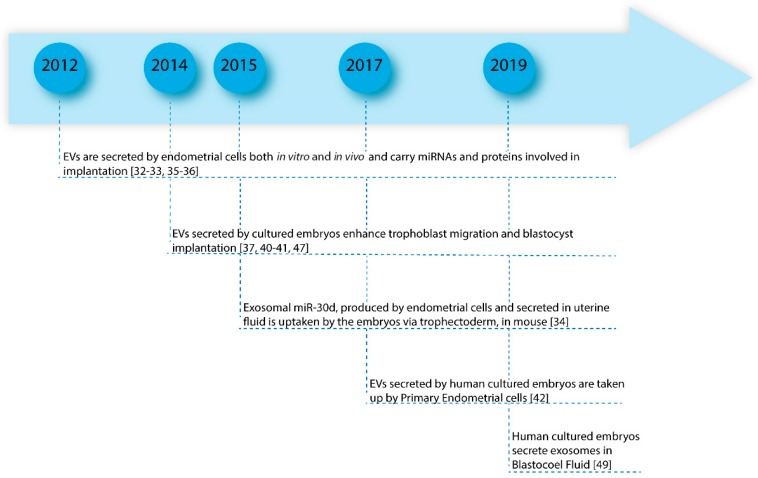
Milestones of the main significant papers about the involvement of EVs in embryo implantation and its early development and their potential role in negative pregnancy outcomes.

In Figure 5 we have reported the milestones of the findings and some of the most significant papers about the involvement of EVs in embryo implantation and its early development and their potential role in negative pregnancy outcomes (Figure 5).

## 4. Conclusions

Surely, in multicellular organisms, EVs enable cellular communication by their cargo and represent biological targets or potential instruments for innovative therapies to be used in different human diseases. In spite of the great expectation, to date different questions remain open. What are the signals that allow the molecules to be enclosed within the vesicles? By which pathway do the cells address MVBs to lysosomes or to the plasmatic membrane? What are the surface markers of the vesicles and the recipient cells? To date, these points have only been partially clarified. Moreover, profiling studies on molecule cargo, certainly are able to identify potential non-invasive biomarkers for specific pathological conditions, but in many cases, we do not know the biological significance of altered expression of different molecules in a specific fluid. Above all, this is true in reproductive biomedicine where the studies of EV role are certainly more recent than in other fields. These observations should lead us to continue and improve basic research focusing our attention to understand the complex mechanisms operating inside the cells.
